# Influence of Parental Physical Activity and Screen Time on the BMI of Adult Offspring in a Saudi Population

**DOI:** 10.3390/healthcare8020110

**Published:** 2020-04-24

**Authors:** Khurshid Mattoo, Mosa Shubayr, Mohammed Al Moaleem, Esam Halboub

**Affiliations:** 1 Department of Prosthetic Dental Sciences, College of Dentistry, Jazan University, Jazan 45142, Saudi Arabia; 2 Department of Preventive and Community Dentistry, College of Dentistry, Jazan University, Jazan 45142, Saudi Arabia; 3 Department of Maxillofacial Surgery and Diagnostic Sciences, College of Dentistry, Jazan University, Jazan 45142, Saudi Arabia

**Keywords:** parenting, obesity, screen time, parental neglect, sharia

## Abstract

Saudi Arabia is witnessing a drastic rise in adult obesity. Geographic limitations hamper somatic activities to counter this rise. Parental physical inactivity in the region has never been addressed. This study’s purpose is to determine the differences between parent and adult child (the subjects here) levels of physical activity (PA) and screen time (ST) between normal weight and obese adults in the Saudi Arabian population. Two hundred and forty adult subjects (18 to 35 years) were screened for their body mass index (BMI) values (18.5 ≤ 25 as normal and 25 ≤ 30) or above as overweight/obese), followed by their congregation into normal weight (N) (*n* = 150) and overweight/obese (Ov/Ob) (*n* = 90) groups. A self-reported questionnaire assessed parenting practices, while a physical activity record diary calculated existing levels of PA and ST. Statistical significance was determined by a chi-squared test (*p* < 0.01) and BMI correlation was found by Pearson’s correlation coefficient. Maternal age (87.8% ≤ 20 years in the Ov/Ob group (Gp) and consanguineous marriage (88.9% in the Ov/Ob Gp) showed significant differences. A high prevalence of inactivity was observed among families (father 53.3%, mother 53.3%, subject 80.0%) in the Ov/Ob Gp. Higher amounts of ST (76.7% ≥ 9 h/day) were found in the Ov/Ob Gp, which significantly differed. Differences in the parent and child levels of PA and ST exist between normal weight and obese Saudi Arabian adults. Physically active parents having adult children inspire them to develop healthy physical behaviors which counter the development of obesity. Consanguineous marriage and early maternal age may be associated with progressive adult obesity.

## 1. Introduction

Slothfulness (“kasal” in Arabic) accounts for laziness or slowness in the mindset of a person. It has been mentioned in three perspectives within the dominion of various religions. These perspectives include physical, mental, and spiritual perspectives. In the present century, the allusion to slothfulness as a cardinal sin among various religions can be justified since such a behavioral disorder has been associated with the progression of childhood and adult obesity. In the scientific world, the term physical inactivity is synonymous with the word sloth, used to describe the environmental influence of developing obesity. Obesity stems from some of the states in the United States of America and has crossed to Europe. Obesity is now even threatening poor nations, especially in the form of major health burdens for country’s average health care costs (0.7% to 2.8%) [1]. The condition received serious scientific attention when its prevalence rose in children, which later was reflected in their adulthood. The etiology of the condition has a multifactorial predisposition. Although between 47% to 80% of individuals have a hereditary (genetic) cause [2], the role of environmental influence in the remaining individuals has directed attention to parenting practices (behavioral, social, and psychological). Investigations into childhood parenting practices have led to the identification of parental neglect [3,4], which subsequently has been accredited into three neglect types, namely, medical, supervisory, and care neglect [5,6]. The progression or regression of obesity from children to adults depends on the habits that are cultivated within a child. Childhood habits develop within the family milieu and tend to be followed by children during adulthood. Besides influencing their children to practice a healthy lifestyle, parents are also accountable to promote or limit any activity that affects their children’s health. A few examples include discouraging skipping meals (feeding habits) [7], limiting screen time (ST) [7], and/or encouraging physical activity (PA) [3]. These three parameters are linked to obesity either directly or indirectly. While some habits can be enforced verbally, others like PA need to be practiced by the parents within the family [8]. Poor role models, especially in the case of the mother, considerably increase the risk of the child to develop adult obesity. The chances of a child being physically inactive increases in parents who are physically inactive, [8,9] especially an inactive mother [10].

An exceptionally alarming rise in obesity (both childhood and adulthood) in the current decade has been reported in economically progressive countries like the Kingdom of Saudi Arabia (KSA). The Kingdom has reported an unprecedented growth in childhood (<18 years) obesity from 3% (1976) to 25%–40% (2014), while, for adulthood (>18 years) obesity, an increase from 20% (2005) to around 66%–75% (2014) has been reported [11,12]. The KSA has a geographic disadvantage (majorly desert) with soaring temperatures and long summers, besides cultural, traditional, religious, and conservative restrictions which restrict PA as a daily routine [13]. Certain marital practices like early female marriage, short intervals between pregnancies, marriages within tribes and relatives, and polygamy among men are unique to the region’s culture, which makes responsible parenting a tedious task, especially for the female gender. The economic prosperity of the region seems to add to the problem, since access to different types of screens influences people to stay indoors. Globally, studies have focused to find the association of parental PA and ST in relation to the age of adolescence, since the maximum influence of parents has been found to occur during adolescence [14]. A recent long term longitudinal study (30 years), however, has found that parental PA is associated with increased levels of children’s physical activity till the age of 24 years, which tends to remain until their midlife, thus making the age category of young adults (18 to 35 years) very significant in tackling adult obesity [15]. In the KSA, it is at this age when most of the people in the region get married and take independent decisions regarding the welfare of their respective families. Additionally, cultural and religious guidelines (Sharia) of Saudi Arabia’s governance demand parental influence to be present throughout life. In the context of this background, this comparative study of the aforementioned age group (18 to 35 years) was therefore considered worth investigating. The study objectively aims to explore the differences in the PA and ST levels between normal weight and obese families (i.e., with the adult child and his/her father and mother) which to our knowledge has not been studied in the KSA. Two novel parenting variables, namely, consanguineous marriage and the parental age at the time of concerned childbirth (normal weight or obese adult) are also investigated for their association with obesity. These two marital variables are unique in this portion of the world, since polygamy induces early marriage among females and at least one or more marriages are with one’s own relatives.

## 2. Materials and Methods

### 2.1. Study Setting, Sample Selection, and Grouping

This comparative study was organized in the southern region of Saudi Arabia between the years of 2018 and 2019. The study sample was chosen from various colleges affiliated to different universities in the region. About 1450 subjects were screened by physicians for their body mass index values (BMI) to prepare a sample of 240 (150 for the normal weight group (N Gp) and 90 for the overweight/obese group (Ov/Ob Gp)) young adults between the ages of 18 and 35 years. BMI was estimated as kilograms (weight) divided by meters (height) squared using standard cutoff points. The sample design for adult subjects in both groups was random sampling (probability sampling) with adequate replacement in both groups. The eligibility criteria for adult subjects to qualify for the study included being a Saudi Arabian national, one or more siblings with a corresponding BMI value (normal weight or overweight), parents not divorced or living separately, both biological parents alive since birth, no history of potential illness of either the parents or subjects, no history of chronic drug use by the parents or by the subject, cooperative parents, no history of recent pregnancy or marriage of the subjects, and no history of substance abuse by anyone in the family. Any subject who was the lone child of his parents, and/or was obese since birth, and/or was obese as a consequence of sudden weight gain, and/or was a single parent orphan was also excluded.

### 2.2. Operational Definition

PA is operationally defined here as a measure of the time (in hours) spent by each individual practicing light, moderate, or heavy exercise other than regular walking during work and/or house maintenance, while ST is defined here as the time (in hours) that an individual spends watching either television, playing or working on a computer/mobile device, or even multi-tasking (using a mobile device while working on a laptop or watching television) per day on average. Decreased physical activity by an individual has been found to be associated with increased time spent on screens, along with the deprivation of a regular sleeping time [16]. The adult subjects of both groups, along with his/her respective parents (father and mother), are denoted as a family for describing the study here.

### 2.3. Socio-Demographic Information and Measuring PA Time

The socio-demographic information for all families in both groups was collected through a written questionnaire. The amount of time spent on PA and ST was gathered using a self-reported activity diary which was provided to all the participants in both groups (Supplementary Materials). The use of standard questionnaires like the International Physical Activity Questionnaire (IPAQ) to collect data related to PA and ST is not preferred, since it introduces recall errors, social desirability bias, and measurement bias. To minimize such errors, a physical activity record diary (BAR) [17] was used here instead, with modification to the number of days. Increasing the number of days resulted in more consistent and reliable data while also excluding any data that had any missing values. The questionnaire to collect demographic data and the physical activity record diary were translated into the native linguistic form using a translation and back translation method [18]. The questionnaires and activity diaries were distributed among all concerned participants during an organized event.

### 2.4. Measures and Statistical Analysis

All families in both groups were requested to record the number of hours they spent on PA and ST every day for a period of two weeks. After the collection of the self-reported questionnaires and activity diaries, the data for each individual were processed, cleaned, and coded for SPSS (Statistical Package for the Social Sciences, version 25, IBM Corp., Armonk, NY, USA) processing (Supplementary Materials). For data analysis, the number of hours spent on PA and ST was kept uniform for all the participants through conversion. The analysis included the determination of absolute and relative frequencies (qualitative variables), mean values ± standard deviation (continuous variables), chi-squared testing (association and/or difference between categorical variables), and Pearson’s correlation coefficient (BMI correlation). All *p*-values were two sided and were considered to be significant when their value was less than or equal to 0.01.

### 2.5. Ethics

The research proposal (No: 37/7/000165) for this study was duly approved by the college and university research committee that conducts all its studies in accordance with Helsinki declaration. Written knowledgeable consent was distributed and acquired from all the participants for the study after clarifying the advantages of the study.

## 3. Results

From a surveyed sample of 1450 subjects, 240 (147 male and 93 female) families fulfilled the eligibility criteria for the study. The sociodemographic comparison of the families in both groups is shown in Table 1. The average age (both groups) of an adult male subject was 22.47 years (the sum of the mean ages in both groups divided by two) and that for female subjects was 21.52 years, respectively. A higher frequency in the average BMI values of the concerned family members (subject, father, and mother) was found for the Ov/Ob Gp than their counterparts in the N Gp. A higher frequency of totally illiterate parents was found in the Ov/Ob Gp than the N Gp. About 77.8% of the subjects in the Ov/Ob Gp gained weight during intermediate/secondary schooling. An average of 74.6% of total participants from both groups, were non-insured for health policies. Table 2 presents the comparative differences among various parenting practice variables that have been found to coincide with the progression of adult obesity. The nurturing maternal variables (i.e., breastfeeding duration, the start of using infant formula, maternal age at child birth (<20 years), and maternal smoking during pregnancy) have demonstrated significant differences (*p* < 0.01) between the two groups. The BMI of mothers was found to be correlated with the subject BMI. The BMI of fathers did not produce any correlation with the BMI of subjects, nor did the paternal age at the subject’s birth or paternal smoking show any significant difference between groups. Culturally unique variables like consanguineous marriage and parental views about a child’s weight both demonstrated significant differences between the two groups. Variables associated with the subject that differed significantly between the two groups included the subjects PA during his schooling and the average sleeping hours/day. The comparative differences in the time spent on the existing PA and ST for families in both groups are presented in Table 3. A higher frequency of parents and subjects in the N Gp spent more time on PA than the Ov/Ob Gp, with the differences between the two being statistically significant (*p* < 0.01). The average ST for parents did not differ statistically, however, the STs for subjects were significantly different between the studied groups.

## 4. Discussion

This study was conducted in order to analyze the differences in the parenting practices between parents of normal weight and overweight adults in a sample of the Saudi Arabian population, whose parenting styles differ from the rest of the world due to cultural, traditional, and religious norms. Adult obesity has experienced an alarming rise in the region, therefore this study differs from the mainstream studies in the age group of the sample that was investigated. The basis of choosing this age group is the claim that the environmental influence of parents differs between childhood and adulthood obesity [15]. While the majority of the parenting variables that we investigated have been substantiated in earlier studies, the principle findings of this study are as follows: The two novel parenting variables that are culturally unique to this region, i.e., the mother’s age at childbirth and consanguineous marriage, showed significant differences between the two studied groups. Since the practice of early female marriage is part of the culture in the Saudi Arabian kingdom, careless parenting may be a probable reason for the surge in the prevalence of adult obesity there. Our data show that 71% (*n* = 90) of subjects’ mothers in the Ov/Ob Gp were married at less than the recommended adult age of 18 years when the concerned subject was born. The tradition of consanguineous marriage has existed in the country for centuries and has possible links to the practice of polygamy. Marriages within families can result in poor parenting practices due to social pressure and expectations from concerned relatives. This can also be a reason for the overpampering of the child by both parents, thus overlooking responsible parenting. The prevalence of consanguineous marriage in Saudi Arabia has been stated to be about 67% (*n* = 3212) [19], and its risk (three times) to develop obesity has also been recently established by Al Harbi et al. in the same population [20]. Our analysis has shown that 52.5% (126 married out of total 240) of parents had a consanguineous marriage among the total subjects, with 88.9% (*n* = 90) being found in the Ov/Ob Gp.

The results of the study also support various views that are associated with the high risk of developing obesity and these include a higher prevalence of obesity being associated with low levels of literacy for parents, [21] a low socioeconomic status, and decreased breastfeeding time with the early introduction and increased consumption of supplemental food while nurturing [22]. Our results also support the general view that breastfeeding is waning in the Arab region [23] among Arab women, which results in an early introduction of formula food. The early feeding of infants (51% by 1 month) which substantially increases (90% by 6 months) has been stated in Saudi Arabia [24]. Our study, however, has found the association of these variables with a different categorization (less than 1 year of age), which coincides with the progression of obesity [25]. Formula food is an energy-dense food which hampers the self-regulation of the child over his energy needs in later life. Irrespective of the child’s feeling of satiety, it is the person holding the bottle who controls the child’s energy intake at a given time [26]. A parent’s opinion regarding his child being overweight is perhaps the first step of recognizing the problem, but 52 percent of parents in the Ov/Ob Gp did not perceive that their child was overweight. Parental perception depends upon their education, resolve to accept, social influences, and their own weight. According to a study, a lower percentage (35%) of parents were able to identify their children as being overweight [27]. Possibly one of the reasons for this parental perception is their cultural views, since the Arab region has been shown to have a preference for a larger body size. Our results show that about 75% of mothers in the overweight Gp were smokers, including the traditional sheesha/hookah and/or cigarettes. Maternal smoking presents a high risk for the development of childhood obesity [28].

Regarding physical activity, while many studies have reported the association between the parental PA and the child/adolescent PA, very few studies has been conducted on the age group that we have investigated here. Since the drastic rise in adult obesity has been reported in the KSA, we believe that when a child has become an adult, the onus is totally on them to be concerned about their own health. Moreover, cultural, governmental, and social outlooks in the kingdom encourage adults to live independently once they get married. Since both the husband and wife are not accountable to their parents for the lifestyle they choose after marriage, this could be one of the reasons that young adults become prone to adopting an inactive lifestyle. Our results show that a higher frequency of physically inactive parents (father 53.3%, mother 53.3%) and inactive subjects (80%) was present in the Ov/Ob Gp as compared to the N Gp. The deposition of body fat is inversely related to PA, which may range from light PA (steps/day) to vigorous PA [29]. Genetic influences on the child’s participation in physical activity [30] and daily physical activity [31] have been recognized, which explains why some subjects are more expected to practice PA than others, and this serves the explanation for the association between parental PA level and offspring BMI. The highest heritability level among different types of activity is that for the level of the inactivity phenotype [32], while studies in accelerometry suggest the heritability of PA to be between the range of 51% to 56% [33]. Body adiposity and PA are also influenced by the environment, especially parental behavior in relation to both parameters. As the child grows, such parental attitudes are cultivated by children and thus persist beyond childhood and adolescent years [32], thus justifying the reason for choosing the age group for this study. Parental influence also differs according to the gender of the child. One child may be influenced by the mother, while the other may be influenced by his/her father. Additionally, child influence may vary according to the conduct of the parents. The frequency of PA by a female child has been shown to be dependent on the type of the family cluster (i.e., obesogenic or non-obesogenic), with decreased PA being observed in an obesogenic cluster at two different periods of times (age of 5 and 7 years) [34]. The idea that the level of parental PA is associated with the PA of the child (3–4 years) is supported through various studies [35], while some studies report to have no association between the two [36,37]. Overall, our study has found that the PA of both parents is significantly associated with the subjects BMI, which is in agreement with other studies [38,39], although the age group here is different from those studies. A study of preschool children, however, did not find any association between maternal PA [40], paternal PA [41], and the child’s BMI. A recent sex specific study of children aged 4–7 years found that leisure time PA was associated with moderate to vigorous PA (MVPA), with maternal PA being significantly related to higher MVPA in girls but not boys, and high paternal MVPA being related to high MVPA in boys [42]. Parental health behaviors including parental weight gain during the offspring’s childhood are risk factors for adulthood obesity [43]. Besides higher parental inactivity, we also found a higher prevalence of physical inactivity (80%) among subjects in the Ov/Ob Gp. A review of physical inactivity levels in Saudi Arabia has shown that the rate of inactivity is in the range of 43.3% to 99.5% [44]. Three studies with different age groups have reported the prevalence of higher inactivity than that in our study (84.7% never active with a mean age of 32.5, 82% never active with a mean age of 43.2, and 88.6% never active with an age range of 16–60 years) [44].

Regarding screen time, no significant differences in the amount of time spent on ST were found among parents of the subjects between two groups. Although, studies have reported that parental time spent on television watching is positively associated with the BMI percentile of their children [45]. The difference in results here may be due to the mode that was investigated, since our study investigated overall ST as compared to only television watching. In the last decade, television programs have become increasingly available on the Internet and are within the reach of every mobile device. Therefore, the evaluation of ST would be more valid than mere television watching. Responsible parenting also demands that parents should have the self-efficacy to limit children’s screen time [46]. The results of our study indirectly indicate that parents in the Ov/Ob Gp cannot efficient to limit the amount of ST of their children. Our results show that about 76% of subjects in the Ov/Ob Gp spend more than 9 h/day on various screens, which is much higher than the maximum recommended time of two hours/day [47]. The results of our study substantiate the previous findings of studies carried out with children aged 8 to 10 years who spend ≥8 h per day [48] and teenagers who spend ≥11 h/day. [49] Our study, however, differs in the age group (18 to 35 years) and the year when the research was conducted. Many social media applications have been launched in the last ten years which are mainly targeted towards youth. The media stats of Saudi Arabia (184.68 cellular subscriptions per 100 people, 98.68% of households have a television, 54 Internet users per 100 people (a total of 9.77 million), and 69.49% fixed Internet broadband subscribers) [50] demonstrate the involvement of the average Saudi Arabian citizen with various types of screens. The economic prosperity of the KSA has not only resulted in the financial growth of its local people, but also has seen a rise in the number of foreigners seeking jobs in the oil producing nation. Such migrant workers in the form of housemaids have been associated with the development of sedentary behavior among locals and arguably has resulted in the development of obesity. The sedentary promoting behavior among local Saudi Arabians has been deliberated by a number of authors. Influences like insufficient knowledge for maintaining a healthy lifestyle [51], nominal school exercise requirements [52], limited access to PA, and, more importantly, conservative social, cultural, and religious norms have been observed. A drastic increase of physical inactivity (84.7% in 1996 to 98.1% in 2011 among women and 43.3% in 1996 to 93.9% in 2011 among men) has also been reported [53]. Digital insurgence and advances in immersive virtual reality games are further reducing PA in children [54].

The relationship between screen time and physical activity has also been explained through various studies. Screen time directly displaces the free time for physical activity [55], where one consumes higher fat diets [56] and also displaces or disturbs sleep patterns [57]. One hour of additional television watching than what is recommended per day increases the prevalence of obesity by 2% [58]. Besides other predictors of adulthood obesity, adolescent weekly screen time independently and significantly predicts the incidence of obesity during adulthood [59]. The relative odds are greater for females (40%) than for males (20%), suggesting gender differences in the association of screen time and the incidence of obesity. The differences have been attributed to the basic biological differences in the energy expenditure in response to screen time and other lifestyle factors [60].

Regarding the limitations of the study, since this study is a cross sectional study, the cause and effect relationship between the variables studied and obesity cannot be concluded. Parenting factors like consanguineous marriage and early parenting should be included in future studies as a possible link to the progression of adulthood obesity. Future studies with a bigger sample size and objectively measured PA are advised. Therefore, further studies need to be carried out in order to overcome these drawbacks. The results for PA and ST obtained in this study were found through a self-entered diary (log), which has the limitation of being burdensome to the participant and inserting any missing data. Bias was minimized but not corrected statistically, therefore inferences should be drawn cautiously. However, cultural limitations that do not allow intervention in the opposite gender prevented objective measurements of PA. Authors should discuss the results and how they can be interpreted in the perspective of previous studies and the working hypotheses. The findings and their implications should be discussed in the broadest context possible. Future research directions may also be highlighted.

## 5. Conclusions

Parents are responsible for developing a healthy lifestyle and practice to effect change in their offspring and play a major role in the prevention of obesity. Adult subjects whose parents were physically active were found to spend more time on physical activity than those whose parents were physically inactive or less active. The amount of time spent on screens by parents is not associated with the adult offspring BMI. However, the time spent on screens by adults was associated with higher BMI values of individuals. Despite the higher ST of subjects in both groups, the time spent in PA is one of the distinguishing features to overcome obesity.

## Figures and Tables

**Table 1:** Sociodemographic comparison between study groups (Normal weight (N Gp) and Overweight/Obese (Ov/Ob Gp).

Characteristic	Categories		NGp (*n* = 150)	Ov/Ob (*n* = 90)
			N (%)	N (%)
Gender	Male		94 (62.7)	53 (58.9)
	Female		56 (37.3)	37 (41.1)
Average age (in years) (mean ± (SD))	Male		22.45 ± 1.39	22.5 ± 1.48
	Female		21.23 ± 1.27	21.82 ± 1.52
Average BMI * (Kg/m2)	Child		22.03 ± 1.28	29.46 ± 2.67
	Father		25.54 ± 1.14	26.03 ± 1.89
	Mother		25.62 ± 1.92	28.37 ± 2.56
Parental Education	Father	Illiterate	23 (15.3)	45 (50.0)
		Up to Secondary school	123 (82.0)	39 (43.3)
		Graduate	4 (2.7)	6 (6.7)
	Mother	Illiterate	52 (34.7)	34 (37.8)
		Up to Primary school	98 (65.3)	56 (62.2)
Parental Occupation	Father	Service	67 (44.7)	14 (15.5)
		Agriculture	37 (24.6)	50 (55.6)
		Business/others	46 (30.7)	26 (28.9)
	Mother	Service	18 (12.0)	4 (4.4)
		Housewife	132 (88.0)	86 (95.6)
Hobbies of the child		Gardening	7 (4.7)	1 (1.1)
		Television	104 (69.3)	76 (84.5)
		Internet	19 (12.7)	8 (8.9)
		Video games	8 (5.3)	2 (2.2)
		Cooking	12 (8.0)	3 (3.3)
Child gaining weight		Primary school	Not applicable	10 (11.1)
		Intermediate/Secondary school		70 (77.8)
		College		10 (11.1)
Health insurance		Yes	40 (26.7)	21 (23.3)
		No	110 (73.3)	69 (76.7)

N = number of participants, SD = standard deviation, BMI = body mass index, % = percentage, * BMI values (<18.5 = underweight, 18.5 to < 24.9 = normal, 25 to < 29.9 = overweight and 30 or higher = obese).

**Table 2:** Comparative differences of environmentally modulated parental variables between study groups.

Parameters	Categories		N Gp (*n* = 150)	Ov/Ob Gp (*n* = 90)	*p*-Value (Chi-Square Test)
			N (%)	N (%)	
Breast feeding of the concerned child	≤1 year		88 (58.6)	90 (100)	<0.01
	≥1 year		62 (41.4)	0 (0.0)	
Introduction of Supplemental foods	≤1 year		126 (84.0)	87 (96.7)	<0.01
	≥1 year		24 (16.0)	3 (3.3)	
Parents respective age at child’s birth	Father	≤30 years	112 (74.8)	49 (54.5)	0.05
		≥31 years	38 (25.2)	41 (45.5)	
	Mother	≤20 years	27 (18.0)	79 (87.8)	<0.01
		≥21 years	123 (82.0)	11 (12.2)	
Parental smoking during pregnancy	Father	Yes	116 (77.3)	88 (97.8)	0.462
		No	34 (22.7)	2 (2.2)	
	Mother	Yes	46 (30.7)	67 (74.4)	<0.01
		No	104 (69.3)	23 (25.6)	
Consanguineous marriage	Yes		46 (30.7)	80 (88.9)	<0.01
	No		104 (69.3)	10 (11.1)	
Sleeping hours of the child (hours per day)	≤8 h		45 (30.0)	87 (96.7)	<0.01
	≥8 h		105 (70.0)	3 (3.3)	
Parents opinion about child weight	Underweight		10 (6.6)	0 (0.0)	<0.01
	Normal		139 (92.7)	52 (57.8)	
	Overweight		1 (0.7)	30 (33.4)	
	Very overweight		0 (0.0)	8 (8.8)	
Childhood physical activity (hours per week)	≤5 h		47 (31.3)	89 (98.9)	<0.01
	6–9 h		92 (61.3)	0 (0.0)	
	≥10 h		11 (7.4)	1 (1.1)	
BMI correlation	Father			0.058	0.368
	Mother			0.316	<0.01 **

* Based on Fisher’s exact test (2-sided), a *p*-value lesser than or equal to 0.01 is considered significant, ** BMI correlation between the child, father and mother was determined using Pearson’s correlation coefficient (*p* < 0.01).

**Table 3:** Comparative differences in physical activity and screen time for parents and child between the two groups and their respective association.

Parameter	Subject	Categories	N Gp (*n* = 150)	Ov/Ob Gp (*n* = 90)	*p*-Value (Chi-Squared Test)
			N (%)	N (%)	
Physical activity *	Father	None/inactive	10 (6.7)	48 (53.3)	<0.01
		≤8 h/week	138 (92.0)	39 (43.3)	
		≥9 h/week	2 (1.3)	3 (3.4)	
	Mother	None/inactive	3 (2.0)	48 (53.3)	<0.01
		≤8 h/week	143 (95.3)	42 (46.7)	
		≥9 h/week	4 (2.7)	0 (0.0)	
	Child	None/inactive	22 (14.6)	72 (80.0)	<0.01
		≤8 h/week	73 (48.7)	18 (20.0)	
		≥9 h/week	55 (36.7)	0 (0.0)	
Screen time **	Father	Less than 3 h/day	29 (19.4)	8 (8.9)	0.028
		3 to 6 h/day	74 (49.3)	45 (50.0)	
		7 to 9 h/day	45 (30.0)	31 (34.4)	
		≥9 h/day	2 (1.3)	6 (6.7)	
	Mother	Less than 3 h/day	12 (8.0)	3 (3.4)	0.05
		3 to 6 h/day	49 (32.6)	28 (31.1)	
		7 to 9 h/day	82 (54.7)	42 (46.7)	
		≥9 h/day	7 (4.7)	17 (18.8)	
	Child	Less than 3 h/day	4 (2.6)	0 (0.0)	<0.01
		3 to 6 h/day	51 (34.0)	5 (5.6)	
		7 to 9 h/day	82 (54.7)	16 (17.7)	
		≥9 h/day	13 (8.7)	69 (76.7)	

* Physical activity of each individual was a measure of how much time was spent other than regular walking during work or house maintenance. It included activities like walking, playing outdoors, gymnasium exercises, and cycling. The categories are developed as per guidelines for physical activity led by the World Health organization. ** Screen time is the amount of time that the subject spends watching television, playing computer/mobile/Internet games and/or multiple tasking (watching television while using mobile) per day on average (to be calculated number of hours per week for statistical consistency).

## Supplementary Materials

The following are available online at https://www.mdpi.com/2227-9032/8/2/110/s1, 1. Raw data 2. Coded data that were submitted during the submission process.
